# Quality of life and functional capacity in depressive patients on hemodialysis: a systematic review and meta-analysis

**DOI:** 10.1590/1414-431X2023e12850

**Published:** 2023-12-18

**Authors:** M.B. Moreira, N.P. Cavalli, N.C. Righi, F.B. Schuch, L.U. Signori, A.M.V. da Silva

**Affiliations:** 1 Programa de Pós-Graduação em Ciências do Movimento e Reabilitação, Universidade Federal de Santa Maria, Santa Maria, RS, Brasil; 2 Programa de Pós-Graduação em Ciências da Reabilitação, Universidade Federal de Ciências da Saúde de Porto Alegre, Porto Alegre, RS, Brasil; 3 Departamento de Métodos e Técnicas Desportivas, Universidade Federal de Santa Maria, Santa Maria, RS, Brasil; 4 Departamento de Fisioterapia e Reabilitação, Universidade Federal de Santa Maria, Santa Maria, RS, Brasil

**Keywords:** Kidney failure, Chronic, Renal dialysis, Depression, Meta-analysis

## Abstract

Depression is a common disorder in patients with chronic kidney disease (CKD), and some data support its relationship with functional capacity and quality of life. However, to date, this has not been evaluated systematically or through meta-analysis. We sought to investigate the relationship of quality of life and functional capacity with depressive disorder in patients with CKD on hemodialysis. This systematic review considered studies published up to 2021 and included cross-sectional and cohort studies. PubMed, Embase, SPORTDiscus, Web of Science, and Cochrane (CENTRAL) databases were used to search for studies. The New Castle-Ottawa Quality Assessment scale was used to measure the quality of the studies. A total of 4,626 studies were found and, after applying the selection criteria, 16 studies (2,175 patients) remained for qualitative analysis and 10 for meta-analysis (1,484 patients). The physical component summary (MD=-6.563; 95%CI: −9.702 to −3.424) and mental component summary (MD=-18.760; 95%CI: −28.641 to −8.879) were lower in depressive patients, as in all Short Form Health Survey 36 (SF-36) domains. Only one study provided data regarding functional capacity, but it was not evaluated by the defined outcome measure. Twelve studies were classified as “moderate quality” (5 to 6 stars) and four were classified as “low-quality” (0 to 4 stars). This meta-analysis with CKD patients on hemodialysis showed a negative relationship between depression and quality of life, with worsening in all physical and mental domains of the SF-36 in depressed patients.

## Introduction

Chronic kidney disease (CKD) is currently a public health problem. In recent years, the number of cases and the number of patients on hemodialysis have increased. There are 2.5 million end stage renal disease (ESRD) patients receiving renal replacement therapy, and this number is expected to increase to around 5 million by the end of this decade (1). In advanced stages of CKD, classified as ESRD, patients are commonly affected by cardiovascular involvement, which is strongly related to the increase in mortality in this population (2).

In addition to cardiac involvement, depression is a common condition in patients with CKD, being identified in 23 to 46% of patients at an advanced stage of the disease (3,4). Some conditions, such as cardiovascular events, number of hospitalizations, physical capacity, and mortality, have been associated with depression (4-6). Data support the relationship between depression and physical capacity, with patients with higher degree of depression having lower functional capacity, as assessed by the 6-minute walk test (6MWT) (5). These data confirm studies that have investigated the effect of exercise on the state of depression in this population (7,8). Furthermore, functional capacity has been shown to be related to quality of life of individuals with ESRD. A systematic review found that elderly patients with CKD have lower levels of quality of life associated with worse functional capacity scores (9).

Previous studies have demonstrated the association between CKD and depression (3,4), emphasizing the importance of this relationship with the progression of the renal pathology and adequate therapeutic management. However, to the best of our knowledge, the influence of depression on functional capacity and quality of life in patients with ESRD has not been shown through systematic review and meta-analysis. Therefore, our study aimed to systematically review the literature investigating the relationship of quality of life and functional capacity with depressive disorder in patients with CKD on hemodialysis.

## Material and Methods

### Study design and ethical issues

This systematic review was conducted in accordance with the recommendations of the Cochrane Handbook, Version 6.1 (10) and with the guidelines suggested by the Meta-Analysis Group of Observational Studies in Epidemiology (11). The protocol was registered in PROSPERO (CRD42021254074) and can be consulted online (https://www.crd.york.ac.uk/prospero/display_record.php?ID=CRD42021254074).

### Inclusion and exclusion criteria

The following inclusion criteria were considered in this systematic review: 1) clinical trials (baseline data only), cohorts, and cross-sectional and case-control trials; 2) studies with adult patients (≥18 years) with ESRD on hemodialysis; 3) studies that assessed the state of depression or major depressive disorder, diagnosed by questionnaires using criteria of Diagnostic and Statistical Manual of Mental Disorders IV (12) or V (13), or by clinicians, psychologists or psychiatrists, or by screening instruments established in the literature (e.g., Hamilton Scale (14), Beck Depression Inventory (15) or others); 4) studies that evaluated at least one of the following two outcomes: a) functional capacity/cardiorespiratory fitness [by the distance walked in the 6MWT or by the direct measurement of VO_2_ max/peak]; b) quality of life [by the Short Form Health Survey 36 (SF-36), 12-Item Short Form Health Survey (SF-12), Kidney Disease and Quality of Life - short form (KDQOL-SF), or other validated instruments that insert variables similar to the others].

The exclusion criteria were studies that evaluated other mental disorders (e.g., bipolar mood disorder, schizophrenia), studies in which patients received other types of renal replacement therapy, conference abstracts, review articles, and articles that were not available in full.

### Search strategy

The search strategy (Supplementary Table S1) were studies published up to December 2021 in the MEDLINE (PubMed), Cochrane CENTRAL, EMBASE, SPORT Discus, and Web of Science databases. In addition, the reference list of included articles or previous reviews were manually searched. Search terms included MeSH terms “Renal Insufficiency, Chronic”, “Kidney Failure, Chronic”, “Kidney Diseases”, “Renal Dialysis”, and “Depression”. The research was carried out without restriction of year of publication or language.

The search strategy was adapted to each database. Two trained reviewers (M.B.M. and N.P.C.) independently selected the studies by title and abstract according to inclusion criteria. Abstracts that did not provide sufficient information for selection were read in full. Subsequently, reviewers evaluated the full texts of potentially relevant studies. Studies that were not fully available were requested from the authors (three attempts, one per week, for three weeks). Any discrepancies were resolved through discussion and consensus or by the decision of a third reviewer (A.M.V.S.). The Mendeley software (https://www.mendeley.com/?interaction_required=true) was used for the study selection process.

### Data extraction and bias assessment

Finally, using standardized forms, two reviewers (M.B.M. and N.P.C.) independently extracted data on study identification, sample size, characteristics (age, sex, body mass index, and educational status), and measuring instruments (presence and/or level of depression, functional capacity/cardiorespiratory fitness, and quality of life). Differences between evaluators were resolved by consensus or by the decision of a third reviewer (A.M.V.S.). When necessary, the first author of the study was contacted for additional information and data.

The methodological quality assessment was performed by two reviewers (M.B.M. and N.P.C.) using the New Castle - Ottawa Quality Assessment Scale, which contemplates three categories (Selection, Comparability, and Exposure) (16). Studies with Newcastle-Ottawa scores (17) ≥7 were considered as high-quality, 5-6 as moderate quality, and 0-4 as low-quality.

### Statistical analysis

Data analysis was performed descriptively and, when appropriate, meta-analyses were performed using the Comprehensive Meta-Analysis Software, version 3 (https://meta-analysis.com). Heterogeneity was assessed using the chi-squared analysis, and the I-squared (I^2^) test indicated low, moderate, and high heterogeneity when I^2^ values were <25%, 25-50%, and >50%, respectively. Meta-analyses were performed for each outcome using the random effect model and mean difference for continuous outcomes. For each of the outcomes, the effect size and its 95% confidence interval (95%CI) were calculated.

## Results

**Figure 1:**
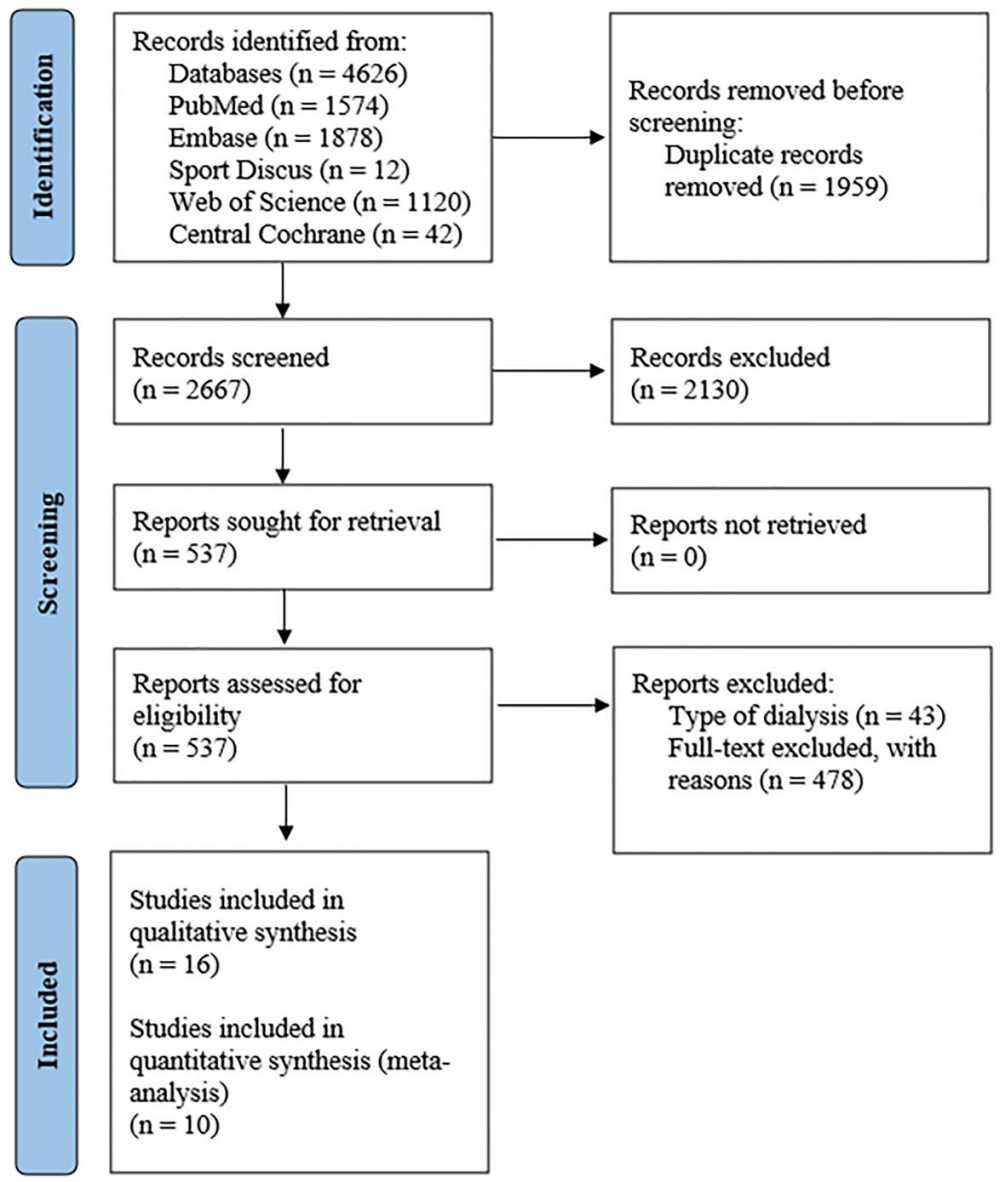
Flowchart of the study.

A total of 4,626 studies were found by searching the databases. After excluding duplicate articles and studies not meeting the inclusion criteria based on titles and abstracts, 537 studies showed potential relevance for full analysis. However, only 16 studies met the predefined eligibility criteria (10 cross-sectional and 6 cohort studies) (Figure 1).

### Study characteristics

The characteristics of the studies included in this systematic review are described in Table 1 (18-33). Regarding the nationality of the studies, a prevalence of Brazil and Taiwan was observed. All articles were published between 2006 to 2021. The total number of patients evaluated in all studies combined was 2,175 subjects.

**Table 1:** Characteristics of studies.

Author (year)	Study design	Country	Sample size (n)	Male (%)	Age (years)	Educational status	Evaluated outcomes
Afsar et al. (2013) 18	Cross-Sectional	Turkey	134	39.5	53±13.4	Primary school: 66 Secondary school: 26 High school: 31 University: 11	Cognitive function, depression, functional capacity, sleep disorders, and quality of life.
Al Zaben et al. (2015) 19	Cohort	Saudi Arabia	40	55	52.1±15.2	6.4±5.0 (n=37)	Cognitive function, depression, physical function, and quality of life.
de Alencar et al. (2020) 20	Cross-Sectional	Brazil	173	58.4	68.7±6.9	Illiterate: 33 1 to 3 years: 41 4 to 7 years: 66 8 or more years: 31	Depression, quality of life.
Barros et al. (2016) 21	Cohort	Brazil	104	59.6	55.3±15.6	NM	BMI, depression, nutrition, and quality of life.
Chen et al. (2010) 22	Cross-Sectional	Taiwan	200	47	58.6±13.9	7.1±4.6	Depression, fatigue, and quality of life.
Cheng et al. (2018) 23	Cohort	Taiwan	151	49	64.6±13.9	≤6 years: 85 >6 years: 66	Cognitive disorders, depression, mortality, and quality of life.
Cruz et al. (2010) 24	Cross-Sectional	Brazil	70	62.9	NM	Up to 4th grade: 9 5th-8th grade:18 1st-3rd grade: 27 College: 16	Depression, quality of life.
de Brito et al. (2019) 25	Cross-Sectional	Brazil	130	52.3	NM	Up to 9 years: 76 10 to 12 years: 32 Over 12 years: 12(n=120)	Anxiety, depression, and quality of life.
Drayer et al. (2006) 26	Cohort	USA	62	51.6	NM	NM	Anxiety, depression, and quality of life.
Ferreira et al. (2011) 27	Cross-Sectional	Brazil	130	63.1	49.7 (18-80)	Illiterate: 21 Elementary: 69 Above elementary: 40	Depression, quality of life.
Garcia et al. (2010) 28	Cross-Sectional	Brazil	47	100	39.4±8.9	NM	Depression, quality of life.
Kardangusheva et al. (2021) 29	Cross-Sectional	Russia	86	70	55.5±13.9	NM	Depression, gender, and quality of life.
Kojima et al. (2010) 30	Cohort	Japan	230	56	56±9.6	Education <12 years: 195 Education ≥12 years: 35	Alexithymia, depression, and quality of life.
Kusztal et al. (2018) 31	Cohort	Poland	203	59.1	60.3±13.8	NM	Depression, pain, and quality of life.
Liu et al. (2017) 32	Cross-Sectional	China	227	53.3	NM	≤Junior high school: 94 >Junior high school: 133	ADLs, depression, and quality of life.
Wang et al. (2014) 33	Cross-sectional	Taiwan	188	47.3	58.5±14	NM	Anxiety, depression, and quality of life.

NM: Not mentioned; BMI: body mass index; ADLs: activities of daily living.

### Main results of the studies

The methods used for assessing depression and quality of life and the main results of the studies are described in Table 2. In most studies, a comparison was made between depressive and non-depressive subjects and the characteristics and associations with several variables, including quality of life, mortality, and physical, cognitive, and metabolic factors.

**Table 2:** Assessment method and main results of the studies.

Author (year)(n=16)	Depression assessment method	Quality of Life assessment method	Main results
Afsar et al. (2013) 18	BDI	SF-36	Patients with internet-based research about their disease had less depressive BDI score (10.77±8.05 *vs* 17.68±10.88, P<0.0001) and had higher SMMSE score (27.3±1.71 *vs* 25.6±1.62, P<0.0001), higher physical functioning (53.3±15.4 *vs* 46.1±13.2, P=0.018), higher role physical limitation (53.1±17.3 *vs* 45.7±14.6, P=0.023), higher general health perception (54.2±13.8 *vs* 47.9±11.2, P=0.019), higher vitality (51.7±12.1 *vs* 46.7±11.6, P=0.031), and higher emotional role functioning (56.8±17.8 *vs* 49.1±16.5, P=0.039) compared to patients who did not have internet based research about their disease.
Al Zaben et al. (2015) 19	HDRS	SF-36	Of the 20 patients with major or minor depressive disorder, eight (40 %) fully remitted by 6 weeks of follow-up, and an additional three patients remitted over the next 6 weeks, leaving 45% with significant depressive symptoms persisting beyond 12 weeks.
de Alencar et al. (2020) 20	GDS	CASP-16	Depression was present in 22.5% of the sample. Depressed patients presented low CASP-16 quality of life scores (31.6 *vs* 24.2, P<0.001).
Barros et al. (2016) 21	BDI	WHOQOL-brief	32 (30.8%) patients had depressive symptoms and a significantly lower QoL compared with the 72 patients in the no depressive symptoms group.
Chen et al. (2010) 22	HADS	SF-36	Depressed patients had greater levels of fatigue (24.7±5.5 *vs* 17.0±4.2, P<0.001) and anxiety (7.7±4.3 *vs* 3.0±2.9, P<0.001) and poorer QoL (36.8±22.7 *vs* 81.9±18.4, P<0.001) than non-depressed patients.
Cheng et al. (2018) 23	TDQ	WHOQOL-brief	Subjects with TDQ scores 19-54 (correlated with clinically significant depressive symptoms) and those with scores 15-18 had higher 3-year mortality rates than the two groups with lower scores (40.0, 46.7, 16.0, and 19.6%, P=0.021, ANOVA).
Cruz et al. (2010) 24	BDI	SF-36 / WHOQOL-brief	Depression prevalence was 14.3% among IHD patients and 9.9% in the hemodialysis group. Depressed patients presented lower QoL scores than non-depressed ones in all domains, and the most affected were emotional role functioning, mental health, and social functioning of SF-36, and psychological domain of WHOQOL-brief.
de Brito et al. (2019) 25	BDI	SF-36	SF-36 mental summary scores were associated with depression among transplantation patients (OR: 0.923; 95%CI: 0.85-0.99; P=0.03) and dialysis patients (OR=0.882; 95% CI: 0.83-0.93; P≤0.001). Physical component summary was associated with depression among dialysis patients (OR=0.906; 95%CI: 0.85-0.96; P=0.001). Summary scores were associated with anxiety among dialysis patients.
Drayer et al. (2006) 26	PHQ9	SF-36 / KDQOL-SF	17 (28%) had major or minor depression. Depressed patients were younger and had lower health-related QoL than did non-depressed patients. Depression predicted mortality (HR=4.1, 95%CI: 1.5-32.2, P<0.05) after adjusting for age, gender, race, medical comorbidities, albumin, kt/V, and/or the presence of diabetes.
Ferreira et al. (2011) 27	BDI	WHOQOL-brief	QoL indexes were better for non-depressed. More differences were observed in the Psychological (Non-depressed: 69.40 and Depressed: 49.22) and Physical (Non-depressed: 62.81 and Depressed: 42.19) Domains; and the Social Relations domain had a better average between the populations, as well as a better correlation with the other domains.
Garcia et al. (2010) 28	HDRS	KDQOL-SF	Depression was observed in 32 (68.1%) patients according to the HDRS. A significant negative correlation was found between the results from the HDRS and the following parameters of the specific dimensions of the KDQOL: list of symptoms and problems (r=-0.399; P=0.005), quality of social interaction (r=-0.433; P=0.002), and quality of sleep (r=-0.585; P<0.001). Among the generic domains, mood showed a significant negative correlation with general health (r=-0.475; P<0.001), emotional well-being (r=-0.354; P=0.015), social functioning, and energy/fatigue (r=-0.518; P<0.001).
Kardangusheva et al. (2021) 29	CES-D	KDQOL-SF	Depression symptoms were detected in 88.4% of patients. Factors have been identified that have a negative impact on QoL: older age, male gender, dialysis experience less than 1 year and more than 5 years.
Kojima et al. (2010) 30	BDI	SF-36	During the follow-up period, 27 deaths were confirmed. Both depression and alexithymia were associated with an increased risk for all-cause mortality. Depression lost its statistical significance after controlling for alexithymia, whereas alexithymia remained significant even after adjusting for the baseline depression, health status (the summary scores of the SF-36), marital status, and clinical covariates (multivariate adjusted HR=3.62; 95%CI: 1.32-9.93; P=0.01).
Kusztal et al. (2018) 31	HADS-D	SF-36	Patients with pain were on maintenance dialysis for longer times and had more depressive symptoms and a lower QoL than those without pain. In the 6-year period, 96 (46.8%) patients died. Highly depressed patients (HADS depression score >8) exhibited higher mortality (<8 *vs* >8 points; P=0.016).
Liu et al. (2017) 32	CES-D	SF-36	The prevalence of depressive symptoms among HD patients was 29.1%. Multivariate logistic regression analyses revealed that ADL (OR=1.124, P=0.002), family support (OR=0.867, P=0.021), “acceptance-resignation” coping style (OR=1.228, P=0.022), and ego resiliency (OR=0.944, P=0.021) were associated with low mood independently.
Wang et al. (2014) 33	HADS-D	SF-36	Forty-five (23.9%) patients fulfilled the DSM-IV-TR criteria for a MDD. The plasma brain-derived neurotrophic factor levels correlated significantly with age and sex but not with depression.

Confidence Intervals (CI); interdialytic weight gain (IDWG); Pittsburgh Sleep Quality Index (PSQI); Beck Depression Inventory (BDI); Standardized Mini Mental State Examination (SMMSE); Short-Form Health Survey (SF-36); Hamilton Depression Rating Scale (HDRS); Geriatric Depression Scale (GDS); Control, Autonomy, Self-realization and Pleasure Questionnaire (CASP-16); World Health Organization Quality of Life instrument-Abbreviated version (WHOQOL-brief); Quality of life (QOL); Comorbidity Index (CCI); Hazard ratio (HR); Hospital Anxiety and Depression Scale (HADS); Taiwanese Depression Questionnaire (TDQ); Ischemic Heart Disease (IHD); Hemodialysis (HD); Odds Ratio (OR); Nine-item Patient Health Questionnaire (PHQ9); Kidney Disease Quality of Life Short Form (KDQOL-SF); Center for Epidemiologic Studies Depression (CES-D); Activities of daily living (ADL); Diagnostic and Statistical Manual of Mental Disorders, Fourth Edition (DSM-IV-TR).

The Beck Depression Inventory (BDI) was the most used instrument to assess depression in the studies. Considering the quality of life assessment, the predominant instruments were the SF-36, followed by World Health Organization Quality of Life instrument-Abbreviated version (WHOQOL-brief) and KDQOL-SF.

### Quality of studies

Supplementary Table S2 shows the quality of the 16 included studies, classified based on the Newcastle-Ottawa Quality Assessment Scale (16). Of the 16 studies, 12 were classified as “moderate quality” (5 to 6 stars) and 4 were classified as “low-quality” (0 to 4 stars).

### Quantitative synthesis (meta-analysis)

**Figure 2:**
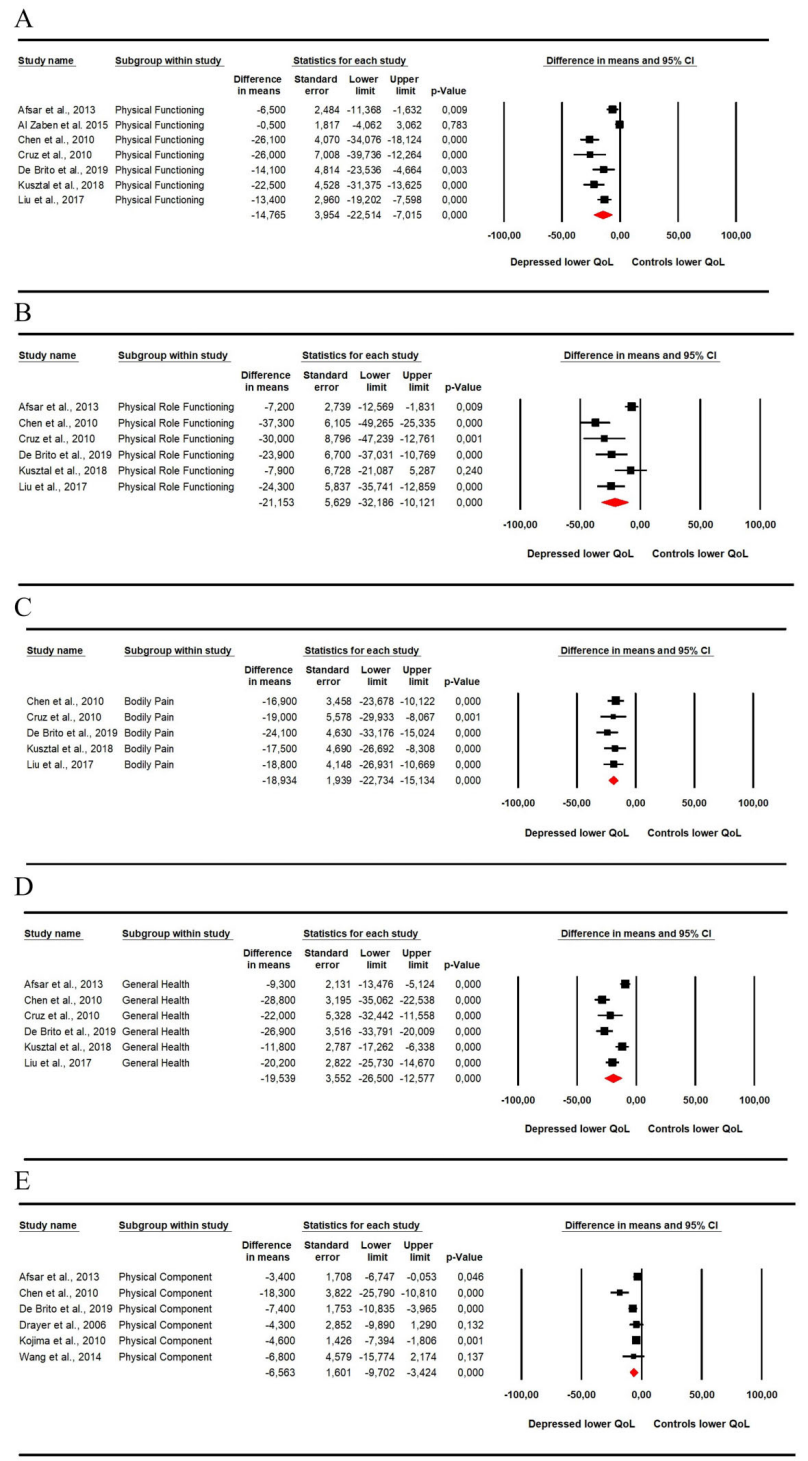
Meta-analysis of the physical aspects of quality of life assessed by the Short Form Health Survey 36 (SF-36), considering the physical functioning (**A**), physical role functioning (**B**), bodily pain (**C**), and general health (**D**) domains, and physical component summary (**E**). Reference numbers can be found in Table 1.

**Figure 3:**
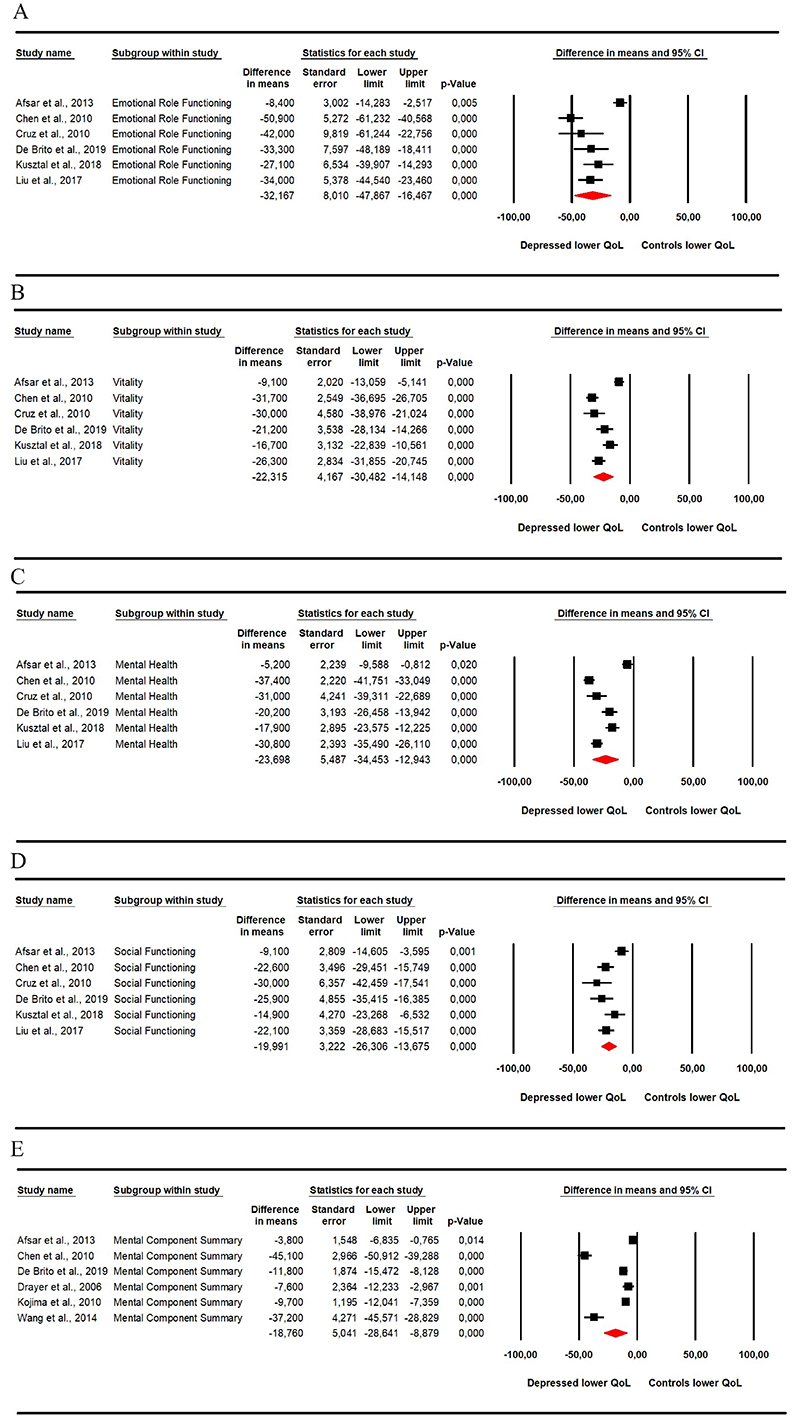
Meta-analysis of the mental aspects of quality of life assessed by the Short Form Health Survey 36 (SF-36), considering the emotional role functioning (**A**), vitality (**B**), mental health (**C**), and social functioning (**D**) domains, and mental component summary (**E**). Reference numbers can be found in Table 1.

A total of 10 studies presented data that enabled quantitative analysis (meta-analysis) of quality of life assessed by the SF-36. The questionnaire has eight scales and two summaries, being divided into physical (Figure 2) and mental aspects (Figure 3). Regarding physical aspects, the physical functioning domain (MD=-14.65; 95%CI: −22.514 to −7.015; Figure 2A) was lower in depressive patients as was physical role functioning (MD=-23.153; 95%CI: −32.186 to −10.121; Figure 2B), bodily pain (MD=-18.934; 95%CI: −22.734 to −15.134; Figure 2C), general health (MD=−19.539; 95%CI: −26.500 to −12.577; Figure 2D), and physical component summary (MD=-6.563; 95%CI: −9.702 to −3.424; Figure 2E).

The analysis of mental aspects also demonstrated a lower quality of life in depressive patients, measured by the emotional role functioning (MD=-32.167; 95%CI: −47.867 to −16.467; Figure 3A), vitality (MD=-22.315; 95%CI: −30.482 to −14.148; Figure 3B), mental health (MD=-23.698; 95%CI: −34.453 to −12.943; Figure 3C), and social functioning (MD=-19.991; 95%CI: −26.306 to −13.675; Figure 3D) domains, and mental component summary (MD=-18.760; 95%CI: −28.641 to −8.879; Figure 3E).

Unfortunately, six studies had insufficient data for quantitative analysis. Three of them provided data from the KDQOL questionnaire and three studies provided data from the WHOQOL-brief questionnaire. In addition, only one study had data regarding functional capacity, which was not evaluated by the distance covered in the 6MWT or by the direct measurement of VO_2_ max/peak, but by the Global Assessment of Functioning (GAF) scale. The results of this study demonstrated a lower functional capacity of hemodialysis patients with depression compared to non-depressive patients. All results showed high heterogeneity considering that I^2^ values were greater than 50% for all domains evaluated.

## Discussion

This study showed a negative relationship between quality of life and depressive disorder in patients with CKD on hemodialysis. The results of this study confirmed the hypothesis that patients with CKD on hemodialysis and depression have a lower quality of life, as observed in all physical and mental domains of the SF-36.

The results of this review are consistent with other studies (20,23), demonstrating the importance of evaluating cognitive and functional factors in patients with ESRD, and that these variables are relevant even in hard outcomes such as mortality (23). The quality of life data presented in our review corroborated the results found in the literature, in which patients with depression on hemodialysis had worse quality of life compared to non-depressive patients (20). Also, in a cohort study with 151 patients, cognitive factors such as depression had a greater influence on quality of life of patients on hemodialysis than somatic factors and were found to be better predictors of mortality over three years (23). These data are consistent with the proposal of the present review to present depression as an independent factor in a population with an important underlying pathology.

The prevalence of depression in this population is well known (34,35). In a cross-sectional study, more than 66% of the evaluated patients had depressive disorders. Furthermore, that study demonstrated an association between depression and social factors (35), which is supported by our findings of impairment in domains related to social and mental function.

AlAwwa et al. (34) demonstrate that hemodialysis may not be associated with a greater or lesser presence of depressive symptoms. However, by including only patients on hemodialysis, our study aimed to homogenize the population studied and, mainly, to identify the influence of depression on the functional capacity and quality of life of patients with ESRD.

No study presented data on functional capacity assessed by maximal or submaximal tests. Only one study evaluated functional capacity (19) by the GAF scale, which made it impossible to assess the variable independently and quantitatively. Even so, our findings were in agreement with hemodialysis patients with depression having lower functional capacity. However, the quality of life of patients was quantitatively evaluated using the SF-36 questionnaire, which includes physical and mental capacity domains. The data from our study corroborated what is reported in the literature (36,37), demonstrating that patients with CKD on hemodialysis and low physical capacity had lower quality of life scores. Differently from the mentioned studies, the present study aimed to demonstrate the importance of depression on physical capacity domains and the influence of CKD on this variable. This is supported by a study in which, among other factors, the association between functional capacity and depression in patients on hemodialysis was identified (5).

Many studies have evaluated different therapies with the aim of improving the functional capacity of patients with CKD, as well as the effect of worsening CKD (38-40). Examples are aerobic and resistance training (38), use of photobiomodulation (39), and neuromuscular stimulation (40). Two recent systematic reviews reported that the implementation of aerobic training associated with resistance training (38) and neuromuscular stimulation (40) improved functional capacity and was associated with improved quality of life of hemodialysis patients. In our quantitative analysis of quality of life, five variables were found to be linked to physical function. Overall, physical function was worse in depressive patients. It is noteworthy that both reviews (38,40) were carried out with the exposure variable (therapy) as the independent variable, unlike the present review in which studies with depression as the independent factor were included.

The methodological quality of the studies was evaluated by the Newcastle-Ottawa Quality Assessment Scale for case control studies (16). Twelve studies had “moderate quality” and 4 studies were classified as “low quality”. The main reasons for these results were the use of self-reported questionnaires, small samples, and the absence of sample calculations. Another point to be considered is the fact that all subjects were recruited in a hospital environment, as both groups (controls and depressives) had underlying disease (ESRD) and required hemodialysis treatment. This can affect the “Selection of Controls” item.

There are limitations in our systematic review that must be considered. Among them, the subjectivity of responses to the depression, quality of life, and referred symptoms questionnaires, as these were self-reported. Because this review included observational studies (cross-sectional and cohort), we cannot confirm these data as they do not provide evidence of cause and effect. Other limitations to consider are the lack of functional capacity data and the high heterogeneity of the studies, which limits the use of more robust methods for the synthesis of results. Due to the small number of studies in each analysis, it was not possible to explore the high heterogeneity with sensitivity and subgroup analyses.

Important data from the analyzed studies, such as educational level, are provided. However, because of the heterogeneous methods of collecting sociodemographic data, it was not possible to carry out a quantitative analysis of relevant variables such as economic and educational status. Several studies confirm the influence of the educational level of patients on therapies related to CKD, as well as on treatment adherence. Another relevant variable for the development of health promotion in this population is the economic level of a country, which reflects the degree of access to possible therapies that patients can use (1). In the qualitative analysis, 16 studies were included, of which 10 were included in the quantitative analysis. These studies are from 9 countries, demonstrating the high heterogeneity of patients and the influence that social and economic factors can have on the outcome of different components of quality of life.

There was a negative relationship between depression and quality of life in patients with CKD on hemodialysis. This finding suggests the need for better assessment (application of diagnostic methods at the right time) and better management of depression. The recognition of depression as an aggravating factor and potential risk to the health of this population is vital for the implementation of multidisciplinary strategies. Prescribing specific therapy to treat depression may lead to improved quality of life and, consequently, better clinical and functional outcomes in these patients.

In conclusion, patients with CKD on hemodialysis and depressive disorder had a lower quality of life compared to non-depressive patients from the same population. This difference was identified in the physical and mental domains of the SF-36. Further studies should compare the functional capacity of depressive and non-depressive subjects with CKD undergoing hemodialysis.

## Supplementary Material

Click here to view [pdf].
